# The ICR142 NGS validation series: a resource for orthogonal assessment of NGS analysis

**DOI:** 10.12688/f1000research.8219.2

**Published:** 2018-09-05

**Authors:** Elise Ruark, Anthony Renwick, Matthew Clarke, Katie Snape, Emma Ramsay, Anna Elliott, Sandra Hanks, Ann Strydom, Sheila Seal, Nazneen Rahman

**Affiliations:** 1 Division of Genetics & Epidemiology, The Institute of Cancer Research, London, UK; 2 Cancer Genetics Unit, Royal Marsden NHS Foundation Trust, London, UK

**Keywords:** Variant calling, next-generation sequencing, NGS, exome, indel, validation

## Abstract

To provide a useful community resource for orthogonal assessment of NGS analysis software, we present the ICR142 NGS validation series. The dataset includes high-quality exome sequence data from 142 samples together with Sanger sequence data at 704 sites; 416 sites with variants and 288 sites at which variants were called by an NGS analysis tool, but no variant is present in the corresponding Sanger sequence. The dataset includes 293 indel variants and 247 negative indel sites, and thus the ICR142 validation dataset is of particular utility in evaluating indel calling performance. The FASTQ files and Sanger sequence results can be accessed in the European Genome-phenome Archive under the accession number EGAS00001001332.

## Introduction

Next-generation sequencing (NGS) approaches have greatly enhanced our ability to detect genetic variation. Over the past decade NGS hardware, software, throughput, data quality and analytical tools have evolved dramatically. Thorough evaluation of each new laboratory and analytical development is challenging but necessary to fully understand how pipeline modification can impact results. To fully assess performance, NGS analysis tools should ideally be run on samples with pre-determined positive and negative sites assessed through orthogonal experimentation such as Sanger sequencing.

Over the past five years, we have generated extensive data on thousands of samples using different NGS instruments, sequencing chemistry, gene panels, exome captures and variant calling tools. Fortuitously, during this process we have generated orthogonal validation data using Sanger sequencing for a core set of 142 samples that were included in the majority of our experiments. We now formally use these samples, which we call the ICR142 NGS validation series, to evaluate NGS variant calling performance after any change to experimental or analytical protocols. This series has proved an extremely useful resource for our assessment of NGS analysis in both the research and clinical settings. We believe that it may also have utility for others, and hence are making it available here.

## Materials and methods

We used lymphocyte DNA from 142 unrelated individuals. All individuals were recruited to the BOCS study and have given informed consent for their DNA to be used for genetic research. The study is approved by the London Multicentre Research Ethics Committee (MREC/01/2/18).

Over the last five years we have generated data from the ICR142 validation series using different exome captures which we have analysed with multiple aligner/caller combinations ^1– 6^. To date we have generated Sanger sequence data for 704 sites amongst the 142 individuals. These sites include variants called by only one aligner and caller combination, increasing the representation of sites which can discriminate performance between methods.

To generate the Sanger sequence data, we performed PCR reactions using the Qiagen Multiplex PCR kit, and bidirectional sequencing of resulting amplicons using the BigDye terminator cycle sequencing kit and an ABI3730 automated sequencer (ABI PerkinElmer). All sequencing traces were analysed with both automated software (Mutation Surveyor version 3.10, SoftGenetics) and visual inspection.

To determine if a variant was present we visually inspected each Sanger sequence with Chromas software v2.13. For each site we selected an ENST from release 65 as the reference sequence. We reviewed at least 100 base pairs of sequence flanking each variant site to allow for position/annotation errors. We considered a base substitution to be confirmed if the correct variant was called at the exact position and the variant base signal was accompanied by a corresponding reduction in the reference base signal. There were 123 confirmed base substitution variants. We considered an indel variant to be confirmed if an indel variant was present in the region of interest and the indel variant allele signal was present along the complete length of the region of interest. There were 293 confirmed indel variants.

**Figure 1.**
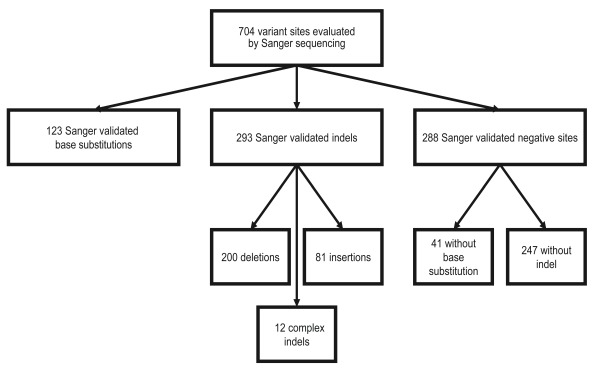
Description of variant sites evaluated by Sanger sequencing in the ICR142 NGS validation series.

We considered a site negative for a base substitution if the specific base substitution was not present, resulting in 41 negative base substitution sites. We considered a site negative for an indel if no indel, of any kind, was detected in the 200 base pair region of interest, resulting in 247 negative indel sites ( Figure 1). We annotated confirmed variants with the HGVS-compliant CSN standard using CAVA (version 1.1.0) according to the transcripts designated in Supplementary table 1 ^7^.

We have also generated high-quality exome sequencing data for the ICR142 NGS validation series. We prepared DNA libraries from 1.5 µg genomic DNA using the Illumina TruSeq sample preparation kit. DNA was fragmented using Covaris technology and the libraries were prepared without gel size selection. We performed target enrichment in pools of six libraries (500 ng each) using the Illumina TruSeq Exome Enrichment kit. The captured DNA libraries were PCR amplified using the supplied paired-end PCR primers. Sequencing was performed with an Illumina HiSeq2000 (SBS Kit v3, one pool per lane) generating 2×101 bp reads. CASAVA v1.8.1 (Illumina) was used to demultiplex and create FASTQ files per sample from the raw base call files.

All of the 704 sites had at least 15× coverage in the exome data, defined as at least 15 reads of good mapping quality (mapping score ≥20). Because these sites are well covered, we can readily assess the variant calling performance of any software tool by applying the pipeline to the exome sequencing data and comparing the variant calls with the Sanger sequencing dataset.

## Data availability

We have deposited the FASTQ files for all 142 individuals in the European Genome-phenome archive (EGA). The accession number is EGAS00001001332.

Researchers and authors that use the ICR142 NGS validation series should reference this paper and should include the following acknowledgement: "This study makes use of the ICR142 NGS validation series data generated by Professor Nazneen Rahman’s team at The Institute of Cancer Research, London”.

## Supplementary material

**Supplementary table 1. Sanger sequencing results for 704 sites in the ICR142 NGS validation series.** Confirmed variants are annotated according to the designated transcript by CAVA using CSN ^7^.

The description of the column headings are given below:

**Sample**          – sample name in the ICR142 series

**Gene**             – HGNC symbol

**SangerCall**    – the most 3’ representation annotated with CSN

**Type**              – “bs”, “del”, “ins”, “complex”, or “indel” for base substitutions, simple deletions, simple insertions, complex indels, or negative indel sites, respectively

**Transcript**     – the ENST ID from Ensembl v65 used to annotate the Sanger call

**CHR**                – chromosome

**EvaluatedPosition** – evaluated hg19 site position, centre of designed amplicon

**POS**              – the left-aligned position in hg19 coordinates for variants called in exome data by Platypus v0.1.5

**REF**              – the reference allele in hg19 for variants called in exome data by Platypus v0.1.5

**ALT**              – the alternative allele in hg19 for variants called in exome data by Platypus v0.1.5

**Zygosity**        – “heterozygous” a variant that is present on one allele, “homozygous” a variant that is present on both alleles.

**SiteID**           – numerical ID (1-704) of sites in the ICR142 NGS validation series
