# Supplementary material for: The ICR142 NGS validation series: a resource for orthogonal assessment of NGS analysis
Source: F1000Res. 2018 Sep 5;5:386. Originally published 2016 Mar 22. [Version 2] doi: 10.12688/f1000research.8219.2 (PMC4857748; doi:10.12688/f1000research.8219.2)
Supplement: Peer review history

# Peer review: Referee response for version 1

**DOI:** 10.5256/f1000research.8841.r13013

**Published:** 2016-05-03

**Authors:** Brad Chapman, Oliver Hofmann

**Affiliations:** 1 Department of Biostatistics, Harvard Public School of Health, Boston, MA, USA; 2 Wolfson Wohl Cancer Research Centre, Institute of Cancer Sciences, University of Glasgow, Glasgow, UK

The authors describe ICR142, a publicly available set of fastq files and confirmed true and false variants for validating analysis pipelines. This is an incredibly useful community resource that complements existing efforts like the Genome in a Bottle project by providing a set of validated, difficult regions to evaluate variant detection tools. I appreciate the efforts to make these test sets public; instead of having validation sets like these developed internally at clinical laboratories, we can collaborate and improve them publicly.

In collaboration with Oliver Hofmann at the Wolfson Wohl Cancer Research Center ( https://twitter.com/fiamh) we obtained access to the data and were able to run a validation using bcbio variant calling ( http://bcbio-nextgen.readthedocs.io). In doing this, we tried to address a couple of challenges for other users wanting to make immediate use of this data in their own in hour validation work:

- The truth sets are not easy to plug into existing validation frameworks. Most validation tools like rtg vcfeval and hap.py work from VCF format files, while this truth set is in a custom spreadsheet format with a mixture of methods for describing changes. You can use Platypus positions for many but need to use CSN descriptions or evaluated position for the remainder.
- The truth sets don't appear to describe if we expect calls to be homozygous or heterozygous calls at each position.
- Many existing validation approaches expect a single (or few) samples so coordinating checking and validation for all these samples can be a challenge.
- As part of this review, we generated a set of configuration files and scripts to help make running validations with ICR142 easier ( https://github.com/bcbio/icr142-validation).

This comparison work also includes a set of comparisons with common callers (GATK HaplotypeCaller, FreeBayes and VarDict). Several of the Sanger validated regions without variants are false positives in at least 2 of the callers tested, so this dataset exposes some common issues with calling and filtering. It would be useful to hear the author's experience with validating callers using this benchmark set and if they have additional filters used to avoid these problems. Knowing a baseline expectation for results would help ensure that the users understand how correctly they've setup the validation resources.

We have read this submission. We believe that we have an appropriate level of expertise to confirm that it is of an acceptable scientific standard.

# Peer review: Referee response for version 1

**DOI:** 10.5256/f1000research.8841.r13347

**Published:** 2016-04-21

**Authors:** Richard Bagnall

**Affiliations:** 1 Agnes Ginges Centre for Molecular Cardiology, Centenary Institute, Sydney Medical School, The University of Sydney, Sydney, NSW, Australia

A myriad of software tools have been developed for the alignment of next generation sequencing data to a reference genome and for the subsequent genotyping of DNA variants. Evaluating the specificity and sensitivity of a variant calling framework can be achieved with a dataset containing validated genotypes. Ruark et. al. provide the ‘ICR142 NGS validation series’ exome sequence fastq files of 142 individuals, and a large set of corresponding Sanger sequencing validated variant sites and sites where variants were called by an NGS tool, but no variant was found with the corresponding Sanger sequencing.

I found the NGS dataset to be easily accessible, on request, from the European Genome-phenome archive and it comprises paired end fastq sequencing files generated by an Illumina sequencing system on the stated 142 individuals. The Sanger sequencing dataset is available as supplementary table 1 of the manuscript. This is a useful resource for evaluating variant calling pipelines.

I have read this submission. I believe that I have an appropriate level of expertise to confirm that it is of an acceptable scientific standard.
